# SPA-UNet: A liver tumor segmentation network based on fused multi-scale features

**DOI:** 10.1515/biol-2022-0685

**Published:** 2023-09-08

**Authors:** Weikun Li, Maoning Jia, Chen Yang, Zhenyuan Lin, Yuekang Yu, Wenhui Zhang

**Affiliations:** School of Computer and Information Security, Guilin University of Electronic Technology, Guilin, Guangxi, 541000, China; School of Business, Guilin University of Electronic Technology, Guilin, Guangxi, 541000, China; School of Information and Communication, Guilin University of Electronic Technology, Guilin, Guangxi, 541000, China

**Keywords:** liver tumor segmentation, dilated convolution, multi-scale, attention mechanism, feature fusion

## Abstract

Liver tumor segmentation is a critical part in the diagnosis and treatment of liver cancer. While U-shaped convolutional neural networks (UNets) have made significant strides in medical image segmentation, challenges remain in accurately segmenting tumor boundaries and detecting small tumors, resulting in low segmentation accuracy. To improve the segmentation accuracy of liver tumors, this work proposes space pyramid attention (SPA)-UNet, a novel image segmentation network with an encoder-decoder architecture. SPA-UNet consists of four modules: (1) Spatial pyramid convolution block (SPCB), extracting multi-scale features by fusing three sets of dilated convolutions with different rates. (2) Spatial pyramid pooling block (SPPB), performing downsampling to reduce image size. (3) Upsample module, integrating dense positional and semantic information. (4) Residual attention block (RA-Block), enabling precise tumor localization. The encoder incorporates 5 SPCBs and 4 SPPBs to capture contextual information. The decoder consists of the Upsample module and RA-Block, and finally a segmentation head outputs segmented images of liver and liver tumor. Experiments using the liver tumor segmentation dataset demonstrate that SPA-UNet surpasses the traditional UNet model, achieving a 1.0 and 2.0% improvement in intersection over union indicators for liver and tumors, respectively, along with increased recall rates by 1.2 and 1.8%. These advancements provide a dependable foundation for liver cancer diagnosis and treatment.

## 1 Introduction

Liver is the largest organ in the human system, with many ducts and a complex anatomical structure. It is located in the right upper abdomen, close to the inside of the ribs. Unlike other organs, the liver has a unique dual blood supply system that comes from the liver’s portal veins (about 3/4) and hepatic arteries (about 1/4). Liver cancer is the most common and deadly tumor in the world, seriously threatening people’s lives and health. According to the National Cancer Center, the incidence of liver cancer in China ranks fifth among all malignant tumors, and the mortality rate ranks second [1]. As science and technology develop, computer application technology and medical information technology are rapidly advancing. Computed tomography (CT) has the properties of fast scanning time and high image resolution and is a common diagnostic method for liver cancer. At present, the segmentation of liver tumors in clinical practice is usually manually marked by experienced physicians, which is not only laborious and time-consuming, but also the tumor area of the liver CT image of the same patient may produce different results when marked by different physicians, which seriously depends on the physicians’ experience and skills. Therefore, it is of great importance to study the accurate and efficient automatic segmentation method of liver tumor for the clinical diagnosis and treatment of liver cancers.

Liver tumors in CT images usually have the characteristics of low contrast, fuzzy boundary, and unfixed shape, size, and number, which lead to inaccurate liver boundary segmentation and difficulty in tumor segmentation. To further increase the accuracy of liver tumor segmentation (LiTS), it is possible to achieve both relatively complete segmentation of larger tumors and detection of smaller tumors. This study proposes a liver tumor space pyramid attention (SPA)-U-shaped convolutional neural network (UNet), consisting of an encoder and a decoder. The multi-scale modules spatial pyramid convolution lock (SPCB) and spatial pyramid pooling block (SPPB) are designed on the encoding path to obtain the multi-scale features of the image by enhancing the receptive field of the segmentation network feature map. Residual attention block (RA-Block) is added to the decoding path, which enables the model to more accurately locate and identify the lesion area. We validate the effectiveness of the designed model on the LiTS task using the public LiTS dataset.

In conclusion, the main work of this study is as follows:

(1) In this study, we propose a network for LiTS, SPA-UNet embedded with SPCB and SPPB, which can extract multi-scale features from images and increase the efficiency of medical image segmentation.
(2) We propose a RA module RA-Block, which can accelerate the training of the network, make the model focus on the region of interest, and suppress the redundant features.
(3) We use the LiTS dataset for experimental analysis, and the results indicate that the network can improve the detection rate of small tumors to some extent, and effectively address the problems of low segmentation accuracy caused by blurred liver tumor borders and the difficulty of tumor segmentation caused by data category imbalance.

The rest of the study is organized as follows: Section 2 introduces the relevant work in this study, Section 3 describes the proposed method, Section 4 presents the experimental results, and Section 5 summarizes the relevant conclusions.

## 2 Recent works

### 2.1 Medical image segmentation

Medical image segmentation is to separate the target region in medical image from the background, usually tumors, organs, and lesions. Medical image segmentation is very challenging, because medical image data usually contains noise, blur, low contrast and other problems, and there are huge changes in the shape and size of the target area in medical images, so efficient and accurate segmentation algorithms are needed. Segmentation of medical images has an extremely significant application value in medical field. It can help doctors diagnose diseases more accurately, make treatment plans, and carry out surgical planning. For example, in the treatment of tumors, the segmentation of medical images can help doctors pinpoint the exact location and extent of the tumor. This enables them to better formulate the treatment plan and predict the effect of the treatment.

However, because of the sheer diversity and complexity of medical image data, segmentation of medical images faces many challenges. For example, there may be plentiful noise and artifacts in medical image data, which has a significant impact on the accuracy of the segmentation algorithm. In addition, different types of medical image data, such as MRI, CT, X-ray, etc., have different characteristics, and the segmentation algorithm needs to be optimized for different types of data. In addition, the shape and size of the target region in medical images change tremendously, which also brings great challenges to the segmentation algorithm.

Traditional methods for medical image segmentation include thresholding [2], level set [3], region growth [4], etc., because liver tumors in CT images usually have the characteristics of low contrast, fuzzy boundary, and uncertain size, shape, position, and quantity, the traditional segmentation methods need manual intervention, which is difficult to effectively adapt to the complexity and diversity of liver tumors, and target segmentation accuracy is low and performance is poor, the automatic segmentation of tumor region cannot be realized.

In recent years, deep learning technology has been under rapid development and is now widely used in the field of medical image segmentation [5–12]. The full convolutional network (FCN) [13] uses end-to-end network to segment medical images. The network classifies images at the pixel level, thus solving the semantic level of image segmentation. The UNet [14] first introduces jump connection into the convolutional network, which realizes image semantic segmentation through encoding-decoding operation. The encoder subsamples the extracted features to capture the image context information. The decoder performs upsampling on the detected features to accurately locate the segmented region. Li et al. [15] proposed a bottleneck supervised UNet. The model is a hybrid tight connection structure, which can be segmented by fully exploiting the information between the layers of the network. Schlemper et al. [16] integrated attention mechanism into UNet and proposed an attention UNet model, which can automatically learn regional features related to segmentation tasks and suppress irrelevant features. Lei et al. [17] proposed a deformable network for liver cancer segmentation. The deformable convolution presented by the network solves the problem of matching irregular liver and liver tumor and enhances feature extraction capability, improving the segmentation accuracy and smoothness of the liver boundary. Zhou et al. [18] improved the jump connection layer of UNet and built a multi-scale UNet network (UNet++) by connecting the jump connections of all layers. Its advantage is that it can extract and integrate features of different scales by superposition. Yang et al. [19] applied UNet++ to liver and tumor segmentation of CT images, and introduced residual structure into the network, effectively solving the problem of gradient dispersion or disappearance in the process of model training. UNet and its variant network [20–24] have been proposed continuously. Based on the above discussion, UNet and its variant network is a high-performance deep learning network that is widely used in medical image segmentation.

However, despite the success of these networks, the local nature of the receptive field in the convolutional layer still limits their learning ability to a relatively small area, which can seriously affect the segmentation performance. Based on this, the dilated convolution used in this work can expand the receptive field of the network to obtain richer local and global context information and improve the segmentation accuracy of the network.

### 2.2 Multi-scale feature fusion

In the task of image segmentation, feature fusion at different scales is an essential method to improve segmentation performance [25–29], and feature fusion can compensate for the lack of pixel values. The low-level features have high spatial resolution and contain more spatial and detailed information, but they have less semantic information and more background noise. The high-level features have stronger semantic information, but they have low spatial resolution and poor perception of detail. Zhao et al. [30] put forward PSPNet, which achieves the prediction effect by fusing different feature layers through pyramid pooling module. Chen et al. [31] proposed that deeplab and its variants use hole convolution with different expansion rates to design ASPP module and fuse multiple feature maps to learn multi-scale features. ASPP aims to enhance the perception of convolutional neural networks for different scales and different semantic information while maintaining resolution. In this article, two multi branch modules, SPCB and SPPB, are designed to fuse multi-scale information to extract features, and attention mechanism is introduced to further improve the network’s feature learning ability for edge and whole tumors.

### 2.3 Attention mechanism

Attention mechanism [32,33,34] is an improved neural network technique proposed in recent years, which has obtained excellent results in the field of image segmentation. The role of the attention mechanism is to make the model focus on more useful semantic information and ignore useless information in order to obtain more global context information, which greatly enriches the representational ability of the neural network. By learning a set of weights, the features of different scales are weighted to improve the response of important features. Dual attention network (DANet) is an attention mechanism proposed by Nanjing University in 2019. It aims to use the attention mechanisms to enhance the perception of feature maps for different locations and different semantic information, and to further improve the performance of semantic segmentation. DANet uses two parallel attention mechanisms to process the feature map, one of which focuses on the correlation between channels, and the other on the correlation between spatial positions. Global context network (GCNet) is a global context attention mechanism proposed by Huawei Noah’s Ark laboratory in 2019, which aims to use global information to enhance the expression ability of local feature maps. GCNet uses the multi-layer global context attention mechanism to process the feature map, respectively, and finally carry out weighted fusion to improve the performance of the model in various computer vision tasks. The advantage of GCNet is that it can take full advantage of global information and does not require additional computation. In addition, GCNet has achieved good performance in many computer vision tasks. For example, GCNet has achieved better results in ImageNet classification tasks. Based on the powerful function of attention mechanism, this study introduces the channel attention mechanism GCT-B0 [35] and coordinate attention (CA) mechanism [28] to improve the network’s segmentation ability for medical images.

## 3 Methods

### 3.1 SPA-UNet architecture

**Figure 1:**
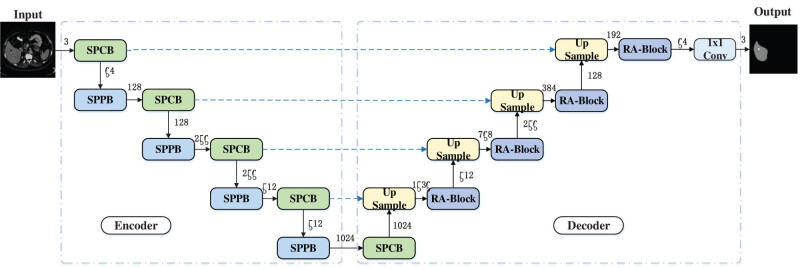
SPA-UNet architecture.

SPA-UNet is a high-precision liver tumor segmentation network designed based on the UNet model, which is structured as shown in Figure 1. The network is made up of an encoder and a decoder, and the encoder is composed of five SPCBs and four SPPBs, where SPCB captures the image context information by three parallel 3 × 3 dilated convolutions. SPPB downsamples the image by switching the 3 × 3 convolution and pooling, which is described in detail in Section 3.3. The number next to each module in Figure 1 indicates the number of channels of the current feature map, so the number of channels of the five feature maps after SPCB are 64, 128, 256, 512, and 1,024, and the amount of channels of the 4 feature maps after SPPB are 128, 256, 512, and 1,024 in order, and the amount of channels of the feature map of the input network after the first SPCB rises from 3 to 64.

The decoder consists of four upsampling structures, Upsample, and four RA mechanism modules, RA-Block. Upsample, while using bilinear interpolation to expand the feature map size to recover the image resolution, is spliced with the corresponding feature maps on the coding path to achieve better feature reconstruction results, combining semantic information with different depths and different fineness in different network layers. RA-Block is a residual structure for accurate tumor localization, and this module is introduced in detail in Section 3.4. As can be seen in Figure 1, the amount of channels of the four feature maps that have gone through Upsample are 1,536, 768, 384, and 192, and the amount of channels of the four feature maps that have gone through RA-Block are 512, 256, 128, and 64 in order, and finally the results of liver and liver tumor segmentations are output by 1 × 1 convolution.

### 3.2 SPCB

**Figure 2:**
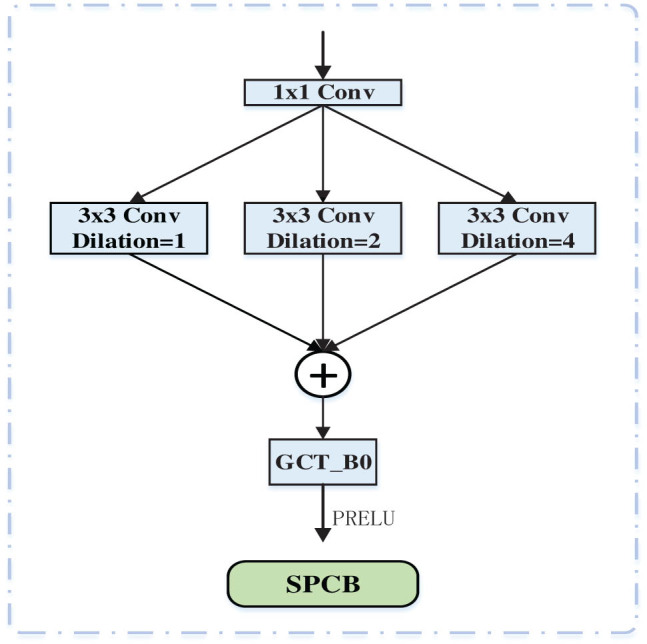
SPCB structure.

To effectively extract multi-scale features from images, we designed the SPCB in the encoder pathway, as shown in Figure 2. In the SPCB component, three parallel dilated convolution sets [36] are utilized with dilation rates of 1, 2, and 4 resulting in respective receptive fields of 3, 5, and 7, which surpasses what can be achieved by standard convolutions. To capture cross-channel information more effectively, the results of the three sets of parallel dilated convolutions are element-wise summed, followed by the Channel Attention Mechanism (GCT-B0). Batch normalization [36] and PReLU activation function [37] are employed to enhance the training process of the network. Furthermore, the SPCB module integrates multiple feature maps instructing the convolutional neural networks to learn multi-scale features capable of enhancing the perception of varying scales and semantic information, while maintaining constant resolution. As a result, the network’s segmentation performance is improved.

Assume that the input of SPCB is *x*(*i*), where *i* denotes the pixel points in the feature map, and the outputs are *y*1(*i*), *y*2(*i*), *y*3(*i*) after three parallel null convolutions, and the expressions are as follows:

(1) \[y1\left(i)\left=\mathop{\sum }\limits_{k=1}^{K}x(i+{r}_{1}\times k\left)\omega \left(k),]\]

(2) \[y2\left({i})\left=\mathop{\sum }\limits_{k=1}^{K}x({i}+{r}_{2}\times k\left){\omega }\left(k),]\]

(3) \[y3\left(i)\left=\mathop{\sum }\limits_{k=1}^{K}x({i}+{r}_{3}\times k\left)\omega \left(k),]\]

where *r* is the dilation rate of the dilated convolution. Since too large a dilation rate for the dilated convolution will lose the image local information and too small a dilation rate will limit the perceptual field size, we set the dilation rates of the three cavity convolutions to *r*_1_ = 1, *r*_2_ = 2, and *r*_3_ = 4.

The three feature maps are then feature added to obtain the output feature map *y*′, with the following expression:

(4) \[{y}^{^{\prime} }={\mathrm{Add}}\left(\mathop{\bigcup }\limits_{k=1}^{3}{y}_{k}(i)\right).]\]

Finally, the feature map *y*′ is batch normalized and PReLU is activated to obtain the output feature map *y*, where the PReLU activation function is given by the following equation:

(5) \[{\mathrm{PReLU}}(x)=\left\{\phantom{\rule[-0.93em]{}{0ex}}\begin{array}{c}x,\hspace{2.5em}{\mathrm{if}}\hspace{1em}x\ge 0\hspace{1em}\\ 0.25x,\hspace{1em}{\mathrm{otherwise}}.\end{array}\right.]\]

### 3.3 SPPB

**Figure 3:**
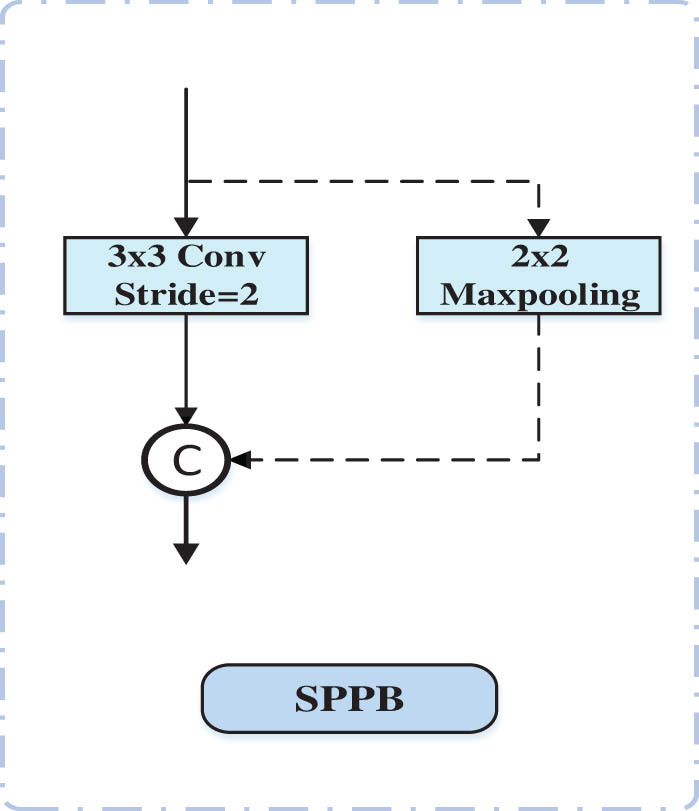
SPPB structure.

The SPPB is used for downsampling the feature maps in the encoder pathway to reduce resolution, as shown in Figure 3. It consists of two branches. The first branch uses standard convolution with a stride of two to reduce the size of the feature maps, while the second branch performs max pooling within non-overlapping 2 × 2 windows. Suppose that the input feature maps have \[{C}_{{\mathrm{in}}}]\] channels, accordingly, the resultant output feature maps will likewise have \[{C}_{{\mathrm{out}}}]\] channels. If \[{C}_{{\mathrm{in}}}\lt {C}_{{\mathrm{out}}}]\] , the max pooling branch will generate feature maps with \[{C}_{{\mathrm{in}}}]\] channels, and the standard convolution branch will generate feature maps with the remaining \[{C}_{{\mathrm{out}}}-{C}_{{\mathrm{in}}}]\] channels. Otherwise, the pooling branch will be ignored, and only the standard convolution branch will be used to generate feature maps with \[{C}_{{\mathrm{out}}}]\] channels.

### 3.4 RA-block

**Figure 4:**
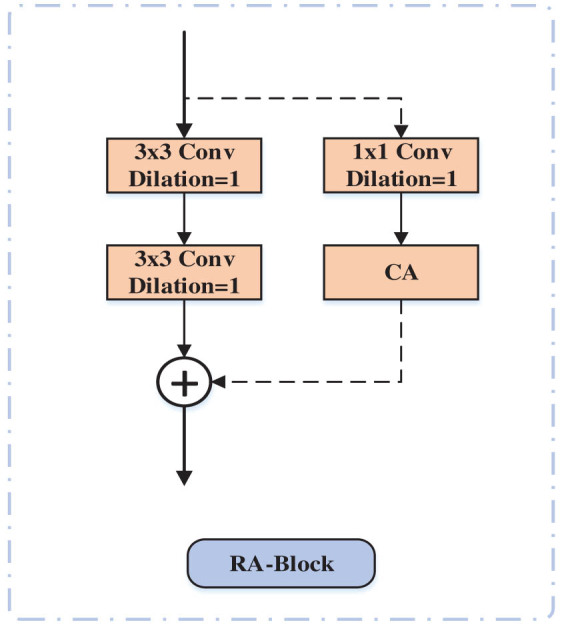
RA-Block structure.

In this study, a RA module (RA-Block) is designed in the decoding path [38], the structure of which is shown in Figure 4. The RA-Block mainly contains two 3 × 3 convolutional layers and a 1 × 1 convolutional and CA layer and performs a feature summation operation. It contains two branches, and for the feature map input to RA-Block, two 3 × 3 ordinary convolution operations are performed on the first branch, and 1 × 1 convolution operations are performed first on the second branch, and the resulting feature map is then passed through the CA layer to make the model focus on the region of interest and suppress redundant features, and finally the feature maps of the two branches are feature summed to form a new feature map as output. This residual module accelerates the convergence of the training network and reduces the model degradation, thus effectively avoiding the gradient disappearance problem.

## 4 Experimental and results analysis

### 4.1 Dataset and preprocessing

In this study, the LiTS dataset was used to train and evaluate the proposed model. The LiTS dataset contains 131 abdominal contrast CT scans with a total of 58,638 CT slices, with the approximate number of CT slices varying from 42 to 1,026, the size of each slice being 512 × 512, and the slice thickness varying from 0.45 to 6 mm. The liver and tumor regions were manually labeled by specialized physicians as the gold standard for segmentation.

**Figure 5:**
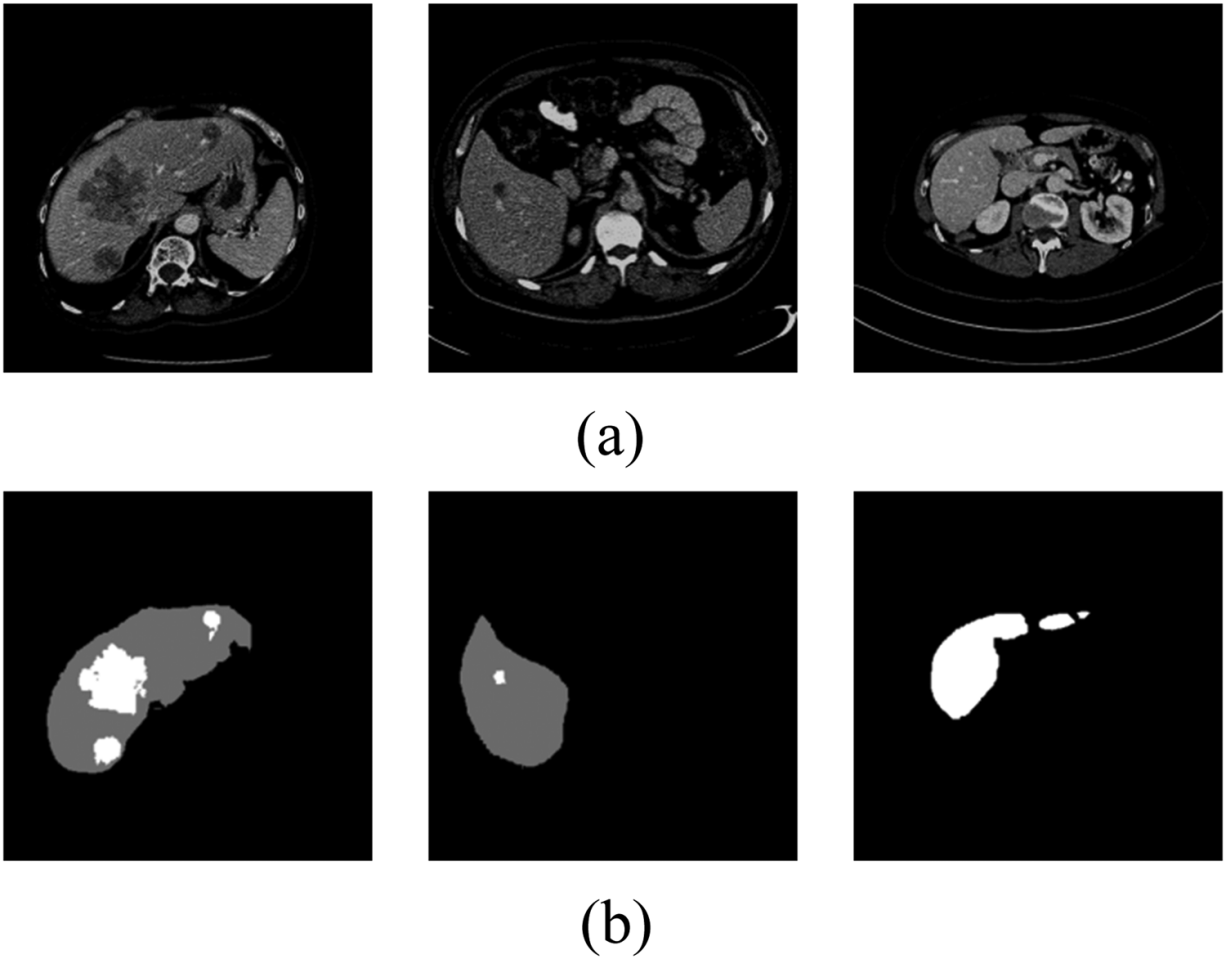
Example of experimental dataset. (a) Abdominal CT image and (b) ground truth for liver tumor segmentation.

Due to the small proportion of liver tumors visible in CT images, inadequate contrast, and indistinct borders, pre-processing the original CT slice is necessary to enhance tumor segmentation accuracy and image clarity. First, in order to increase the contrast of liver tissue and exclude the interference of other organs, the window width and window position of CT images are set to 200 and 60 Hu, respectively, in this study. Then, the CT slices without liver labels are removed because the dataset contains images of multiple organs of the abdomen, but only the liver and liver tumors are segmented in this study. After the dataset was preprocessed, the count of CT image slices per patient varied from 28 to 312, as a result, there exists an aggregate of 19,211 CT slices each measuring 512 × 512 in size, and randomly divided into 15,367 slices for the training set and 1,922 slices each for the validation and test sets. Some sample examples are shown in Figure 5.

### 4.2 Evaluation metrics

To validate the effectiveness of the proposed model in this study, commonly used medical image evaluation metrics including Intersection over Union (IoU), Precision, and Recall were employed. The formulas for these metrics are as follows:

(6) \[\text{IoU}=\frac{\text{TP}}{\text{TP}+\text{FN}+\text{FP}\hspace{.25em}},]\]

(7) \[\text{Precision}=\frac{\text{TP}}{\text{TP}+\text{FP}},]\]

(8) \[\text{Recall}=\frac{\text{TP}}{\text{TP}+\text{FN}}.]\]

The term True positive “TP” corresponds to accurately predicted positive samples. These positive samples correspond to areas where the predicted output overlaps with manually annotated ground truth data. False positive (FP) represents the incorrectly predicted positive samples, which are the regions predicted as positive but not present in the ground truth; False negative (FN) represents the incorrectly predicted negative samples, which are the regions present in the ground truth but not predicted as positive. The values of these three metrics range from 0 to 1, where a value closer to 1 indicates a better segmentation performance, as it indicates a closer resemblance between the predicted results and the ground truth.

### 4.3 Experimental setup

The experimental configuration comprises a single Tesla V100 GPU loaded with 32 gigabytes of dedicated video memory. Additionally, it features a high-performance Gold Intel processor consisting of 24 cores. The software environment used was Ubuntu 16.04, Python 3.7.4, and the deep learning framework used was PaddlePaddle 2.4.0 with GCC version 7.3.0. The experimental parameters are listed in the table. Data augmentation techniques, including random scaling, random horizontal flipping, random padding and cropping, and random distortion, were applied during the network training process to enhance the model’s robustness. (Table 1)

**Table 1:** Experimental parameters

Parameter name	Parameter selection
Optimizer	SGD
Learning rate	0.01
Weight delay	4 × 10^−5^
Momentum	0.9
Batch size	4
Epoch	50
Loss	Cross-entropy

### 4.4 Comparative experiments

#### 4.4.1 Liver image segmentation

To demonstrate the segmentation performance of the proposed SPA-UNet model, we compared it with several state-of-the-art models, including UNet, TopFormer [39], SegFormer_B0 [40], BiSeNet V2 [41], FCN, OCRNet [42], Deeplabv3, UNet++, Attention UNet, ESPNet [43], PSPNet, etc. The performance of these different networks in liver segmentation based on the three evaluation metrics is shown in Table 2.

**Table 2:** Performance comparison of quantitative metrics for liver segmentation with different networks

Model	IoU	Precision	Recall
UNet	0.943	0.961	0.966
TopFormer	0.903	0.943	0.956
SegFormer_B0	0.943	0.969	0.973
BiSeNetV2	0.932	0.963	0.967
FCN	0.955	0.978	0.972
OCRNet	0.874	0.921	0.944
Deeplabv3	0.955	0.977	0.977
UNet++	0.942	0.966	0.971
Attention UNet	0.952	0.971	0.977
ESPNet	0.925	0.961	0.961
PSPNet	0.955	0.979	0.975
Ours	0.953	0.974	0.978

According to the results shown in Table 2, it can be seen that the proposed SPA-UNet model exhibits superior performance compared to the original UNet model in terms of evaluation metrics. Specifically, SPA-UNet achieves an improvement of 1.0, 1.3, and 1.2% for IoU, Precision, and Recall, respectively, when compared to the original UNet model. Furthermore, compared to OCRNet, SPA-UNet shows improvements of 7.9, 5.3, and 3.4% in IoU, Precision, and Recall, respectively. However, FCN, Deeplabv3, and PSPNet slightly outperform SPA-UNet in certain metrics, as these three models have been pretrained with pretrained weights, while SPA-UNet is trained from scratch. Overall, the proposed SPA-UNet method demonstrates superior performance compared to other networks in liver segmentation task, achieving higher accuracy.

#### 4.4.2 Liver tumor segmentation

Table 3 displays the performance results of various neural network architectures for liver tumor segmentation, assessed using the three evaluation metrics.

**Table 3:** Performance comparison of quantitative metrics for liver tumor segmentation with different networks

Model	IoU	Precision	Recall
UNet	0.806	0.885	0.889
TopFormer	0.662	0.805	0.787
SegFormer_B0	0.792	0.884	0.883
BiSeNetV2	0.751	0.875	0.841
FCN	0.818	0.896	0.903
OCRNet	0.732	0.859	0.832
Deeplabv3	0.832	0.910	0.906
UNet++	0.796	0.883	0.890
Attention UNet	0.821	0.908	0.896
ESPNet	0.774	0.874	0.820
PSPNet	0.827	0.903	0.907
Ours	0.826	0.902	0.907

From Table 3, we can see that the model proposed in this work improves 2.0, 1.7, and 1.8% in IoU, Precision, and Recall indexes, respectively, relative to the original UNet, and 14.4, 8.0, and 10.2%, respectively, relative to TopFormer. This result proves the effectiveness of the proposed module, indicating that fusing multi-scale features and increasing the perceptual field of the network is beneficial to the extraction of fine details of boundaries and deeper small structure features. This improves the model’s ability to learn features, while the attention mechanism used in this study allows the model to focus on more effective features and to suppress irrelevant features, resulting in overall better performance on the LiTS task than other networks, and the segmentation of tumors is more accurate, which is an essential reference for the diagnosis of liver cancer.

### 4.5 Results visualization

**Figure 6:**
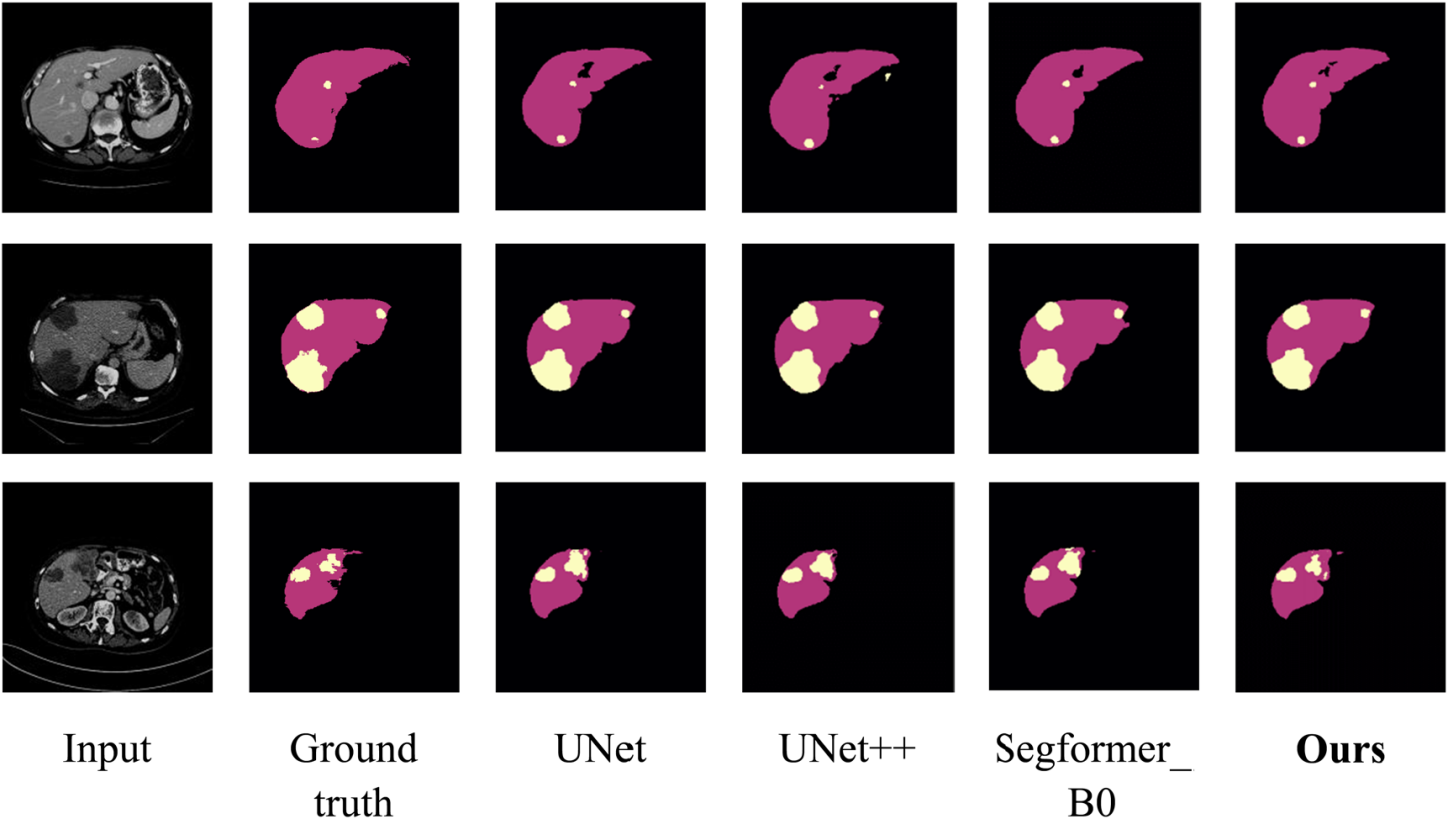
Visual comparison of liver tumor segmentation results with different networks.

To compare the segmentation power of the proposed model with that of other networks such as UNet, UNet++, and SegFormer_B0, Figure 6 illustrates visualized segmentation results for liver tumor segmentation tasks. The first column displays preprocessed abdominal CT slice images, the second column presents corresponding liver tumor segmentation ground truth labels, and the subsequent four columns illustrate the predicted segmentation outcomes by utilizing UNet, UNet++, SegFormer_B0, and our proposed model, respectively. In these images, red regions denote liver segments while yellow regions indicate tumor segments.

Figure 6 indicates that our proposed method generates segmentation results that are more similar to the ground truth compared to other networks such as UNet and UNet++. Compared to these models, our method delivers smoother liver edge segmentation, and for tumor segmentation, it can accurately identify both large and small tumors, effectively addressing issues of under-segmentation and over-segmentation. This improved performance can be attributed to the introduction of residual modules in SPA-UNet, which helps to capture fine details at the edges during the segmentation process. The adoption of dilated convolutions in the network also enables the extraction of rich features, including high-resolution liver edges and complete tumor information. Additionally, the channel attention mechanism and CA mechanism capture important feature information in both spatial and channel dimensions, resulting in more accurate segmentation of liver tumors.

### 4.6 Ablation experiments

#### 4.6.1 Impact of different dilation rates in SPCB

As observed from Tables 4 and 5, using three different dilation rates of 1, 2, and 4 in the atrous convolutions in the SPCB improves the segmentation performance of the model. Lower or higher dilation rates result in decreased segmentation accuracy. A smaller dilation rate may cause the network to overly focus on local details while neglecting contextual information at larger scales, whereas a larger dilation rate may lead to the loss of regional information. Therefore, in this study, we designed the SPCB with three different dilation rates of 1, 2, and 4.

**Table 4:** Impact of different dilated rates on liver segmentation

Dilation	IoU	Precision	Recall
(1,2,2)	0.952	0.975	0.976
(2,2,4)	0.952	0.975	0.976
(2,4,4)	0.952	0.974	0.976
(1,2,4)	0.953	0.974	0.978

**Table 5:** Impact of different dilated rates on tumor segmentation

Dilation	IoU	Precision	Recall
(1,2,2)	0.823	0.900	0.906
(2,2,4)	0.824	0.908	0.899
(2,4,4)	0.824	0.907	0.899
(1,2,4)	0.826	0.902	0.907

#### 4.6.2 Impact of channel attention mechanism in SPCB.

As per Tables 6 and 7, it is evident that SPCB with GCT-B0 demonstrated higher performance metrics compared to the model without GCT-B0. Specifically, the IoU metric showed improvements of 0.2 and 0.3%, and the Recall metric showed improvements of 0.1 and 1.2%, respectively. However, the Precision metric showed a decrease when using SPCB with GCT-B0. This is because Recall and Precision are mutually influenced, and a higher Recall rate can result in lower Precision. Therefore, this study embeds GCT-B0 in the SPCB to better improve the regulation of the feature channels in the encoder part of the model.

**Table 6:** Impact of channel attention mechanism on liver segmentation

Attention	IoU	Precision	Recall
NULL	0.951	0.973	0.977
GCT-B0	0.953	0.974	0.978

**Table 7:** Impact of channel attention mechanism on tumor segmentation

Attention	IoU	Precision	Recall
NULL	0.823	0.910	0.895
GCT-B0	0.826	0.902	0.907

#### 4.6.3 Impact of each branch in SPPB

The SPPB has two branches, and since the pooling operation cannot change the number of channels, this part of the ablation experiment uses a convolution with a stride of two for the downsampling operation. According to the experimental results, it can be seen that the downsampling effect of the spliced convolutional and pooling layers is better and can reduce the loss caused by downsampling. Because the convolution with a step size of two expands the receptive field while performing downsampling, better feature reconstruction results are achieved. (Tables 8 and 9)

**Table 8:** Impact of each branch on liver segmentation

Branch	IoU	Precision	Recall
Conv	0.831	0.904	0.911
Conv+pool	0.953	0.974	0.978

**Table 9:** Impact of each branch on tumor segmentation

Branch	IoU	Precision	Recall
Conv	0.473	0.791	0.540
Conv+pool	0.826	0.902	0.907

#### 4.6.4 Impact of CA mechanism in RA-block.

After adding the CA mechanism after the 1 × 1 convolutional layer of the original residual structure, the segmentation performance is improved, highlighting the edge features of large targets and the global features of small targets, solving the original UNet information redundancy transfer problem, and improving the IoU index by 0.2 and 0.6% on the liver and the tumor segmentation tasks, respectively, thus adding the CA mechanism to SPA-UNet. (Tables 10 and 11)

**Table 10:** Impact of CA mechanism on liver segmentation

Attention	IoU	Precision	Recall
NULL	0.951	0.974	0.976
CA	0.953	0.974	0.978

**Table 11:** Impact of CA mechanism on tumor segmentation

Attention	IoU	Precision	Recall
NULL	0.820	0.908	0.895
CA	0.826	0.902	0.907

## 5 Conclusion

This work proposes a liver tumor segmentation network based on the fusion of attention mechanism and multi-scale features. SPCB and SPPB are designed on the coding path to extract the multi-scale features of the image, capture the context information, and introduce the channel attention mechanism GCT into SPCB_B0 to enable the model to capture the important characteristic information of spatial dimension and channel dimension. The RA module is introduced into the decoding path to speed up the network’s convergence speed, focus the model on the region of interest, and suppress redundant features. The experimental results show that, compared with UNet and other advanced medical image segmentation networks, the overall performance of this method is better than other networks, and it has achieved good results in the task of liver tumor segmentation, and has strong robustness. However, in practical application, there are problems such as large workload of labeling samples, high cost of network calculation, and difficult training. In the next work, we will develop a lighter network model and maintain the accuracy of the segmentation, so that it can be better exploited for the adjunctive liver cancer diagnosis or in other clinical scenarios.
